# Bifenthrin Diminishes Male Fertility Potential by Inducing Protein Defects in Mouse Sperm

**DOI:** 10.3390/toxics12010053

**Published:** 2024-01-10

**Authors:** Jeong-Won Bae, Ju-Mi Hwang, Minjung Yoon, Woo-Sung Kwon

**Affiliations:** 1 Department of Animal Science and Biotechnology, Kyungpook National University, Sangju 37224, Gyeongsangbuk-do, Republic of Korea; jwbae1822@gmail.com (J.-W.B.); ghkdwnal100@gmail.com (J.-M.H.); mjyoon@knu.ac.kr (M.Y.); 2 Research Institute for Innovative Animal Science, Kyungpook National University, Sangju 37224, Gyeongsangbuk-do, Republic of Korea

**Keywords:** bifenthrin, differentially expressed sperm proteins, sperm dysfunction

## Abstract

A synthetic pyrethroid pesticide, bifenthrin, has been commonly used as an effective exterminator, although the rise in its usage has raised concerns regarding its effects on the environment and public health, including reproduction, globally. The current study investigated the function-related molecular disparities and mechanisms in bifenthrin-exposed sperm cells and the underlying mechanism. Therefore, epididymal spermatozoa were released, and various concentrations of bifenthrin were treated (0.1, 1, 10, and 100 μM) to evaluate their effects on sperm. The findings showed that although bifenthrin had no effect on sperm viability, various other sperm functions (e.g., motility, spontaneous acrosome reaction, and capacitation) related to male fertility were decreased, commencing at a 1 µM treatment. Molecular studies revealed nine differentially expressed sperm proteins that were implicated in motile cilium assembly, sperm structure, and metabolic processes. Furthermore, bifenthrin affected sperm functions through abnormal diminution of the expression of specific sperm proteins. Collectively, these findings provide greater insights into how bifenthrin affects male fertility at the molecular level.

## 1. Introduction

Synthetic pyrethroid pesticides have been commonly used as effective exterminators of undesirable organisms in urban and agricultural applications for several decades. Pyrethroids are derivatives of a natural compound, pyrethrin, which is isolated from *Chrysanthemum cinerariaefolium* [1]. They can be classified into types I and II based on the presence of α-cyano. Both types predominantly target the nervous system by inhibiting voltage-gated sodium channels and delaying closure; however, the changes induced in the sodium channels differ between type I and II pyrethroids [2,3]. Furthermore, several pyrethroids act on gamma amino butyric acid-gated chloride and calcium channels [4].

To date, several studies have examined the negative effects of pyrethroids (e.g., neurotoxicity) on nontarget organisms [5,6]. Several studies have demonstrated that aquatic organisms are the most severely impacted organisms by pyrethroids [7,8], whereas another study reported that mammals exhibit lessened ion channel sensitivity and relatively reduced absorption through the skin compared with insects [9], resulting in lower pyrethroid toxicity. In humans, pyrethroids are rapidly metabolized through hydrolysis and oxidation [10]. Owing to these characteristics, pyrethroid pesticides, such as bifenthrin, have become increasingly popular.

Bifenthrin is a type I (i.e., non-cyano) pyrethroid and one of the most commonly used insecticides globally [11]. In the United States, the annual usage of bifenthrin has been shown to exhibit a rising trend, with the estimated agricultural use in 2018 being approximately 1.5 million pounds [12]. However, this widespread use has resulted in nontarget organisms being unintentionally exposed to bifenthrin through various routes, including soil, groundwater, and daily intake [13,14], raising concerns regarding environmental contamination and global public health. Bifenthrin has also been detected in human urine samples from Poland [15] and breast milk samples from Brazil, Colombia, and Spain [16].

Several studies have examined the effects of bifenthrin on the reproductive system, with Inland Silverside, also known as *Menidia beryllina*, previously shown to exhibit decreased expression of choriogenin protein when exposed to 5 ng/L bifenthrin. Furthermore, the quantity of fertilized eggs decreased upon exposure to 0.5 ng/L bifenthrin, in line with the downregulation of endocrine-related gene expression [17]. Mauduit et al. [18] reported that exposure to 2, 10, and 100 ng/L bifenthrin after fertilization resulted in the embryos of longfin smelt (*Spirinchus thaleichthys*), exhibiting smaller hatchlings and yolk sac volumes. 

Bifenthrin toxicity to the mammalian reproductive system has also been examined previously, with exposure to 10 µM bifenthrin resulting in altered expression of implantation-related genes and chemokines, which play a key role in the interactions between the uterus and embryo in porcine trophectoderm and endometrial luminal epithelial cell lines [19]. Additionally, in vitro assays have shown that bifenthrin inhibits the expression of LH/hCG-induced ovulatory genes (e.g., Ptgs2) in ovarian granulosa cells of rats [20]. An in vivo study conducted by Zhang et al. [21] showed that bifenthrin decreased the weight of the seminal vesicles, Cowper’s gland, and glans penis in male rats. Furthermore, another study reported that exposure to bifenthrin (5 mg/kg body per day) for 35 days decreased the sperm count and increased the proportion of sperm with abnormal morphologies such as disorganized seminiferous tubules [22]. In addition, bifenthrin significantly altered the PKA activity and tyrosine phosphorylation, which are the key steps of capacitation [23]. Consequently, unintentional exposure to bifenthrin can induce sperm cell dysfunction [21,22,23] and may contribute to male factor infertility. However, the precise molecular mechanisms underlying the toxic effects of bifenthrin on sperm remain unclear. The current study proposed the hypothesis that bifenthrin induces molecular disparities in certain pathways in sperm, which in turn suppress male fertility. Therefore, this study aimed to evaluate the molecular processes and pathways in bifenthrin-exposed sperm and comprehensively examine their effects on sperm function.

## 2. Materials and Methods

### 2.1. Chemicals and Media

Modified Tyrode’s medium (containing 97.84 mM NaCl, 1.42 mM KCl, 0.47 mM MgCl_2_·6H_2_O, 0.36 mM NaH_2_PO_4_·H_2_O, 5.56 mM D-glucose, 25 mM NaHCO_3_, 1.78 mM CaCl_2_·2H_2_O, 24.9 mM Na-lactate, 0.47 mM Na-pyruvate, 2 µg/mL gentamycin, and 0.005 mM phenol red) was used as a basic medium (BM). To induce sperm capacitation, 0.4% bovine serum albumin (BSA) was added to the BM. Unless otherwise stated, all chemicals and reagents were obtained from Sigma-Aldrich, St Louis, MO, USA.

### 2.2. Sample Collection and Treatment

ICR male mice (Nara Biotech, Seoul, Republic of Korea) were kept individually (12-h light/dark cycle, 22 ± 2 °C with 40–60% humidity) and provided ad libitum water and feeding (Cargill Agri purina, Inc., Seongnam, Republic of Korea). Epididymal spermatozoa were collected from 19 mice, aged 10–11 weeks and released into BM containing 0.4% BSA following the standard procedure as described previously [23,24]. The samples were then incubated with various concentrations of bifenthrin (0.1, 1, 10, and 100 μM) at 37 °C under 5% CO_2_ for 90 min. The optimal concentrations of bifenthrin were considered based on previous findings [19,20,25].

### 2.3. Sperm Motility and Kinematics

Sperm motility and kinematic parameters were evaluated using computer-assisted sperm analysis system, CASA, FSA2016 (Medical supply, Seoul, Republic of Korea), OLYMPUS BX43 phase-contrast microscope (Olympus, Tokyo, Japan), and CMOS CAMERA with 2048 × 1536 (300 M pixel) and 60 Frame (Medical supply). Sperm samples were transferred to a Makler counting chamber (Sefi-Medical Instruments, Haifa, Israel), which was warmed at 37 °C. At least 300 sperm per treatment group were then evaluated. The parameters examined included total sperm motility (MOT, %), progressive sperm motility (PRG, %), hyperactivated sperm motility (HYP), curvilinear velocity (VCL, μm/s), straight-line velocity (VSL, μm/s), average path velocity (VAP, μm/s), linearity (LIN = VSL/VCL × 100, %), straightness (STR = VSL/VAP × 100, %), beat cross frequency (BCF, Hz), and amplitude of lateral head displacement (ALH, µm).

### 2.4. Capacitation Status

The capacitation status was assessed using a combined Hoechst 33258 (H33258) and chlortetracycline (CTC) fluorescence staining method. Following the induction of capacitation, 135 µL of the sample was incubated with 15 μL of H33258 solution (10 μg H33258/mL PBS) for 10 min at room temperature (RT). Subsequently, 250 µL of a 2% (*w*/*v*) polyvinylpyrrolidone solution was added; the sample was washed and resuspended in 100 μL of PBS, and 100 μL of CTC solution [750 mM CTC in 5 μL buffer (20 mM Tris, 130 mM NaCl, and 5 mM cysteine, pH 7.4)] was added. After incubating for 20 min at 4 °C, an OLYMPUS BX43 epifluorescence illumination with ultraviolet excitation/emission filters (BP 340–380/LP 425 and BP 450–490/LP 515, Olympus) was used to evaluate the capacitation status. Finally, 400 spermatozoa per treatment group were categorized into three groups based on their capacitation status (i.e., AR pattern = acrosome reacted, green fluorescence appeared only in the midpiece and tail regions; B pattern = capacitated, green fluorescence above the acrosome region with a dark band at the post-acrosome region; F pattern = non-capacitated, green fluorescence all over the sperm).

### 2.5. Intracellular ATP Levels and Cell Viability

The intracellular ATP levels and cell viability were analyzed using ATP assay (Abcam, Cambridge, UK) and cell cytotoxicity assay (Abcam) kits. The assays were conducted following the manufacturer’s instructions, and the absorbance of the intracellular ATP and cell viability were recorded using GloMax Discover instrument (Promega, Madison, WI, USA).

### 2.6. Two-Dimensional Electrophoresis

Differentially expressed proteins (DEPs) were identified using two-dimensional electrophoresis. For protein extraction, the samples (50 × 10^6^ /mL) were incubated in a rehydration buffer at 4 °C for 1 h [7 M urea, 2 M thiourea, 4% CHAPS (*w*/*v*), 0.05% Triton X-100, 24 μM PMSF, 1% octyl β-d-glucopyranoside, 20 mM DTT, 0.5% IPG buffer, and 0.005% bromophenol blue]. Subsequently, 250 µg of solubilized protein was applied to Immobiline DryStrip (pH 3–11 NL, 24 cm; Cytiva, Marlborough, MA, USA) for 12 h at 4 °C. Isoelectric focusing was performed following voltage steps: 100 V for 1 h, 200 V for 1 h 500 V for 1 h, 1000 V for 1 h, 5000 V for 1.5 h, 8000 V for 1.5 h, and finally 8000–90,000 V for 1 h. The obtained strips were first equilibrated with equilibration buffer A [6 M urea, 75 mM Tris-HCl (pH 8.8), 30% (*v*/*v*) glycerol, 2% (*w*/*v*) SDS, 0.002% (*w*/*v*) bromophenol blue, and 2% (*w*/*v*) DTT], and then with equilibration buffer B [6 M urea, 75 mM Tris-HCl (pH 8.8), 30% (*v*/*v*) glycerol, 2% (*w*/*v*) SDS, 0.002% (*w*/*v*) bromophenol blue, and 2.5% (*w*/*v*) iodoacetamide]. Second-dimension electrophoresis was performed using 12.5% (*w*/*v*) SDS-PAGE gels, and the strips were run at 100 V for 1 h and 500 V until the bromophenol blue reached the end of the gel. Subsequently, the gels were silver stained as per the manufacturer’s instructions (Amersham Biosciences, Piscataway, NJ, USA). The molecular weights ranged from 6.5 to 200 kDa, and the pH levels ranged from 3 to 11. A GS-800 calibrated Imaging Densitometer (Bio-Rad) was used to perform spot comparison and identification of the gels in each group. Finally, the numerical values representing protein expression levels were calculated using PDQuest 8.0 software (Bio-Rad, Hercules, CA, United States).

### 2.7. Protein Identification

#### 2.7.1. In-Gel Digestion

The proteins were subjected to in-gel trypsin digestion. The excised gel spots were destained using 100 μL of a solution containing 30 mM potassium ferricyanide and 100 mM sodium thiosulfate and shaken for 5 min. After removal of the destaining solution, the gel spots were incubated with 200 mM ammonium bicarbonate for 20 min, subjected to dehydration using 100 μL of acetonitrile, and dried in a vacuum centrifuge. This dehydration and drying process was repeated three times. The dried gel pieces were then rehydrated with 20 μL of 50 mM ammonium bicarbonate solution containing 0.2 μg of modified trypsin (Promega) for 45 min on ice, after which 70 μL of 50 mM ammonium bicarbonate was added to the gel pieces. Enzymatic digestion was performed overnight at 37 °C, and the resulting peptide solution was desalted using a home-made C18 nanocolumn.

#### 2.7.2. Desalting and Concentration

Custom-made chromatographic columns were utilized for the desalting of the peptide mixture prior to mass spectrometric analysis. The column consisted of 100–300 nL of Poros reverse-phase R2 material (bead size of 20–30 μm, PerSeptive Biosystems) within a constricted GELoader tip (Eppendorf, Hamburg, Germany). Gentle air pressure was applied via a 10 mL syringe to facilitate liquid flow through the column. Subsequently, 30 μL of the peptide mixture from the digested supernatant was diluted in 30 μL of 5% formic acid, loaded onto the column, and washed with an additional 30 μL of 5% formic acid. For tandem mass spectrometry (MS/MS) analysis, the peptides were eluted using 1.5 μL of a solution containing 50% methanol, 49% H_2_O, and 1% formic acid.

#### 2.7.3. Liquid Chromatography MS/MS (LC–MS/MS)

The samples were resuspended in a solution of 0.1% formic acid in distilled water for use with the Ultimate 3000 (Thermo Fisher Scientific, Inc., Waltham, MA, USA). An autosampler was employed to inject 2 μL aliquots of the peptide solution into a C18 column (75 μm × 15 cm, particle size 2 μm) at a flow rate of 300 nL/min. Mobile phase A consisted of 0.1% formic acid in DW, and mobile phase B contained 0.1% formic acid in 90% acetonitrile. The liquid chromatography gradient elution began at 5% to 95% mobile phase B within 47.5 min and held at 95% for 5 min. Finally, it returned to 5% mobile phase B for an additional 5 min. The mass spectrum (MS) was scanned within the range of 150−2000 *m*/*z*.

#### 2.7.4. Database Search

An MS/MS ion search was performed using MASCOT software (version 2.4.1, Matrix Science, Boston, MA, USA), and peptide fragment data were acquired from the peptide peaks in ESI-MS through ESI-MS/MS. Trypsin was selected as the enzyme of choice, allowing for a maximum of two potential missed cleavage sites. The instrument type was ESI-TRAP, and the peptide fragments were searched against the database using MASCOT software (version 2.4.1, Matrix Science) and FASTA search engine. The search was limited to Mus musculus taxonomy within the NCBInr and UniprotKB databases. Mass tolerance was set to ± 10 ppm for peptides and ± 0.8 Da for fragments, and high-scoring peptides were defined as those with a score greater than the default significance threshold in MASCOT (*p* < 0.05, peptide score > 55).

### 2.8. Western Blot Analysis

DEP validation was conducted using Western blot analysis. The samples (50 × 10^6^ /mL) were lysed using modified Laemmli sample buffer (315 mM Tris, 10% glycerol, 10% sodium dodecyl sulfate, 5% 2-mercaptoethanol, and 5% bromophenol blue), and the lysates were separated using 12% SDS–PAGE (Mini PROTEIN Tetra Cell, Bio-Rad) before being transferred onto Immun-Blot polyvinylidene difluoride membranes (Bio-Rad), which were then incubated with 3% ECL blocking agent (GE Healthcare, Chicago, IL, USA) at RT for over 2 h. Subsequently, the membranes were washed with DPBS containing 0.01% Tween-20 (PBST) and incubated with primary antibodies, which were appropriately diluted in 3% ECL blocking agent (GE Healthcare). The proteins were randomly selected from two functionally related groups, cilium and metabolism [AKAP4 Polyclonal Antibody (1:2000; MyBioSource), ATP Synthase O Polyclonal Antibody (1:3000; Invitrogen), C6orf206 Polyclonal Antibody (1:300; Invitrogen, Thermo Fisher Scientific, Inc., Waltham, MA, USA), FABP9 Monoclonal Antibody (1:1000; Invitrogen), and SPA17 Polyclonal Antibody (1:3000; Invitrogen)], and an anti-⍺-tubulin mouse antibody (1:5000; Abcam) was used as a loading control. After treatment with the primary antibody, the membranes were washed with PBST. Next, the membranes were treated with anti-rabbit IgG, HRP-linked antibody (1:2000; Cell Signaling Technology, Danvers, MA, USA), or goat anti-mouse IgG H&L (HRP) (1:2000; Abcam), all of which were diluted in 3% ECL blocking agent (GE Healthcare). The bands were measured using the iBright CL1500 imaging system (Invitrogen) with an ECL substrate (Bio-Rad). Finally, the protein expression signals were quantified using Image Lab software (version 6.1.0, Bio-Rad) and normalized to those of the control.

### 2.9. Functional Annotation, Signaling Pathways, and Protein–Protein Interaction Networks of DEPs

To elucidate the molecular signaling pathways and functional properties, biological process (BP), cellular component (CC), and molecular function (MF) of the DEPs were annotated using the Gene Ontology (GO) databases via Database for Annotation, Visualization, and Integrated Discovery (DAVID 2021) [26] and Enrichr [27]. Furthermore, based on the GO database, pathway enrichment analysis was conducted using ShinyGO 0.77 [28], and the protein–protein interaction networks were constructed using the latest version of STRING (12.0) (http://string-db.org; accessed on 5 September 2024).

### 2.10. Statistical Analysis

All data management and statistical analyses were performed using SPSS software (Version 26.0, IBM, Armonk, NY, USA). To assess the impact of bifenthrin on sperm function, one-way ANOVA was employed. Additionally, the correlation between sperm functional parameters and protein expression was evaluated using Pearson correlation coefficients. Numerical data were presented as mean ± standard error of the mean, and the level of significance was set to *p* < 0.05 for all analyses.

## 3. Results

### 3.1. Sperm Motility and Kinematics

The current study evaluated changes in three motility parameters and seven kinematic parameters in the control and treatment groups. As shown in Table 1, none of the parameters exhibited significant decreases at the lowest bifenthrin dose (0.1 µM) of bifenthrin, whereas significant reductions in MOT, PRG, and LIN were observed in the groups treated with bifenthrin doses of ≥1 µM (*p* < 0.05). Furthermore, all kinematic parameters, excluding LIN, exhibited significant decreases only at the highest concentration of bifenthrin (100 µM; *p* < 0.05), with hyperactivated sperm motility being decreased over 50% compared to the control. Collectively, these findings suggest that bifenthrin at concentrations of ≥1 µM induced significant reductions in sperm motility and kinematic parameters.

### 3.2. Capacitation Status

**Figure 1:**
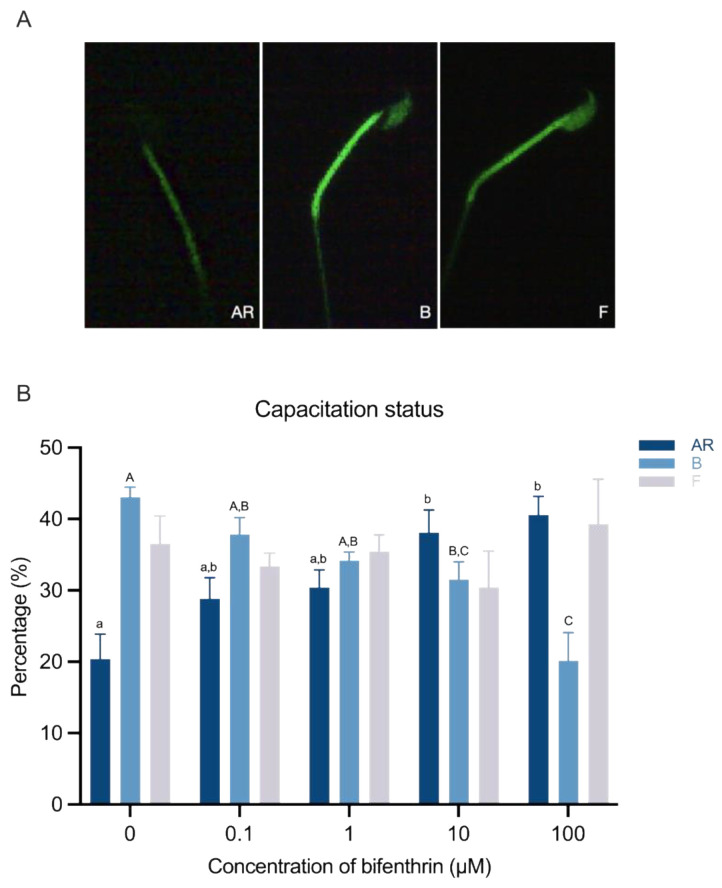
Alterations in sperm capacitation status induced by bifenthrin treatment. Percentage of altered capacitation status visualized in histogram. (**A**) The representative fluorescence image showing the difference status of capacitation pattern. AR = acrosome reacted sperm, green fluorescence appeared only in the midpiece and tail regions; B = capacitated sperm, green fluorescence above the acrosome region with dark banded at the post acrosome region; F = non-capacitated sperm, green fluorescence all over the sperm. (**B**) Indigo bar = AR pattern; sky-blue bar = B pattern; light-gray bar = F pattern. Data represent mean ± SEM, *n* = 4. Superscript letters (a, b, A, B, and C) indicate significant differences between control and each treatment group (*p* < 0.05).

The capacitation status was categorized into three distinct groups. The percentages of AR patterns increased aberrantly at treatment concentrations of ≥10 µM (*p* < 0.05), with the highest concentration (100 µM) resulting in a >2-fold increase compared with the control group (*p* < 0.05). In contrast, the percentages of pattern B showed a significant reduction at treatment concentrations of ≥ 10 µM, with the highest concentration resulting in a more than 50% reduction compared with the control group (*p* < 0.05). However, no significant differences were observed in the F pattern. Overall, 10 µM bifenthrin treatment resulted in abnormal alterations in sperm capacitation status (*p* < 0.05; Figure 1).

### 3.3. Intracellular ATP Level and Cell Viability

**Figure 2:**
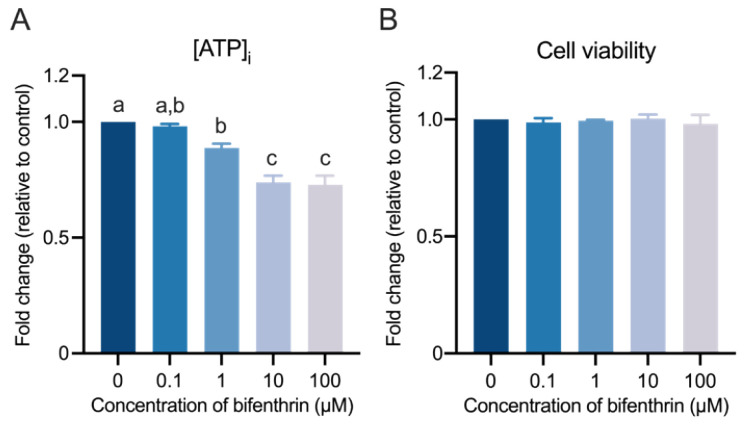
Effects of bifenthrin on intracellular ATP levels and cell viability. Histogram presents the bifenthrin-affected intracellular ATP levels and cell viability of fold change relative to control. (**A**) The effects of bifenthrin on intracellular ATP levels were measured. Data represent mean ± SEM, *n* = 5. Superscript letters (a, b, and c) indicate significant differences between the control and treatment group (*p* < 0.05). (**B**) The effects of bifenthrin on cell viability.

The results of intracellular ATP levels and cell viability are depicted in Figure 2. Intracellular ATP levels exhibited a significant decrease following the administration of 1 µM bifenthrin treatment in comparison to the control group (*p* < 0.05; Figure 2A). Conversely, no difference in cell viability was observed between control and treatment groups (Figure 2B). These results indicate that bifenthrin leads to a reduction in intracellular ATP levels without eliciting differential effects on sperm cell viability.

### 3.4. Differentially Expressed Proteins

**Figure 3:**
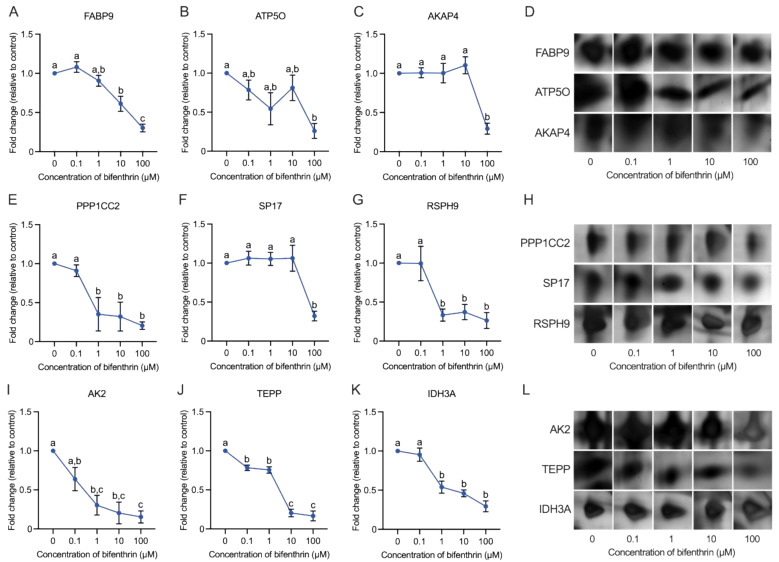
Inhibitory effect of bifenthrin on sperm protein expression. Alterations in bifenthrin-affected sperm protein expression are visualized in line graph. Data are presented as mean ± SEM, *n* = 3. Values with different superscripts (a, b, and c) indicate significant differences between control and each treatment group (*p* < 0.05). (**A**) Fold-change in FABP9 expression levels. (**B**) Fold-change in ATP5O expression levels. (**C**) Fold-change in AKAP4 expression levels. (**D**) Representative protein spots of FABP9, ATP5O, and AKAP4. (**E**) Fold-change in PPP1CC2 expression levels. (**F**) Fold-change in SP17 expression levels. (**G**) Fold-change in RSPH9 expression levels. (**H**) Representative protein spots of PPP1CC2, SP17, and RSPH9. (**I**) Fold-change in AK2 expression levels. (**J**) Fold-change in TEPP expression levels. (**K**) Fold-change in IDH3A expression levels. (**L**) Representative protein spots of AK2, TEPP, and IDH3A.

To evaluate the effects of bifenthrin on sperm proteins, the changes in protein expression levels in the control and treatment groups were examined. As shown in Table 2, a total of nine DEPs [fatty acid-binding protein 9 (FABP9); ATP synthase subunit O, mitochondrial (ATP5O); A-kinase anchor protein 4 (AKAP4); isoform 2 of Serine/threonine-protein phosphatase PP1-gamma catalytic subunit (PPP1CC2); sperm surface protein Sp17 (SP17), radial spoke head protein 9 homolog (RSPH9); adenylate kinase 2, mitochondrial isoform b (AK2); testis, prostate, and placenta-expressed protein isoform 2 (TEPP); isocitrate dehydrogenase (NAD) subunit alpha, mitochondrial (IDH3A)] were identified. As illustrated in Figure 3, the expression of all nine proteins was drastically diminished. Thus, based on the data shown in Figure 3D,H,L, it is apparent that the DEP spots exhibited a clear decreasing trend compared with the control group. TEPP expression decreased significantly at the lowest concentration of bifenthrin (0.1 µM; *p* < 0.05; Figure 3J), whereas PPP1CC2, RSPH9, AK2, and IDH3A expression decreased significantly at concentrations of >1 µM. Among the four proteins, the expression of PPP1CC2, RSPH9, and IDH3A plateaued at treatment concentrations of ≥1 µM (*p* < 0.05; Figure 3E,G,I,K), whereas FABP9 expression decreased in a dose-dependent manner upon exposure to ≥10 µM bifenthrin (*p* < 0.05; Figure 3A). Finally, the expression of ATP5O, AKAP4, and SP17 decreased only at the highest concentration of bifenthrin (100 µM; *p* < 0.05; Figure 3B,C,F).

### 3.5. Verification of the DEPs 

**Figure 4:**
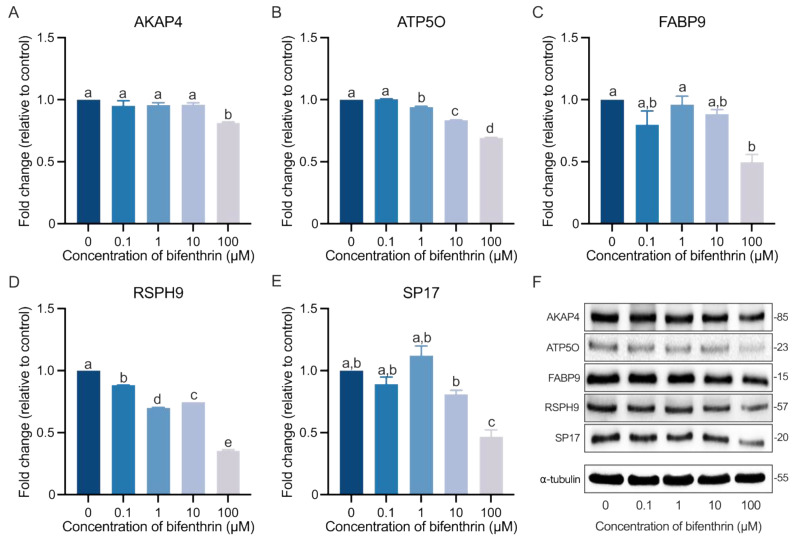
Verification of the expression level of DEPs. The fold-change in protein expression levels of five randomly selected DEPs was verified and shown in the histogram. Data are presented as mean ± SEM, *n* = 3. Superscript letters (a, b, c, d, and e) indicate significant differences between control and each treatment group (*p* < 0.05). (**A**) Fold-change in AKAP4 expression levels. (**B**) Fold-change in ATP5O expression levels. (**C**) Fold-change in FABP9 expression levels. (**D**) Fold-change in RSPH9 expression levels. (**E**) Fold-change in SP17 expression levels. (**F**) The representative protein bands of AKAP4, ATP5O, FABP9, RSPH9, SP17, and ⍺-tubulin were detected approximately at 85, 23, 15, 57, 20, and 55 kDa, respectively.

The DEPs were validated using Western blot analysis (Figure 4). All protein bands exhibited a clear decreasing trend (Figure 4F), with RSPH9 expression levels being significantly decreased at the lowest concentration of bifenthrin (0.1 µM; *p* < 0.05; Figure 4D). Proteins ATP5O and SP17 exhibited reduced expression at concentrations of 1 and 10 µM, respectively (*p* < 0.05; Figure 4B,E). Conversely, AKAP4 and FABP9 expression decreased significantly only at the highest concentration of bifenthrin (100 µM; *p* < 0.05; Figure 4A,C). These findings revealed that even the lowest concentration of bifenthrin can affect protein expression in mouse spermatozoa.

### 3.6. Bioinformatics Analysis

**Figure 5:**
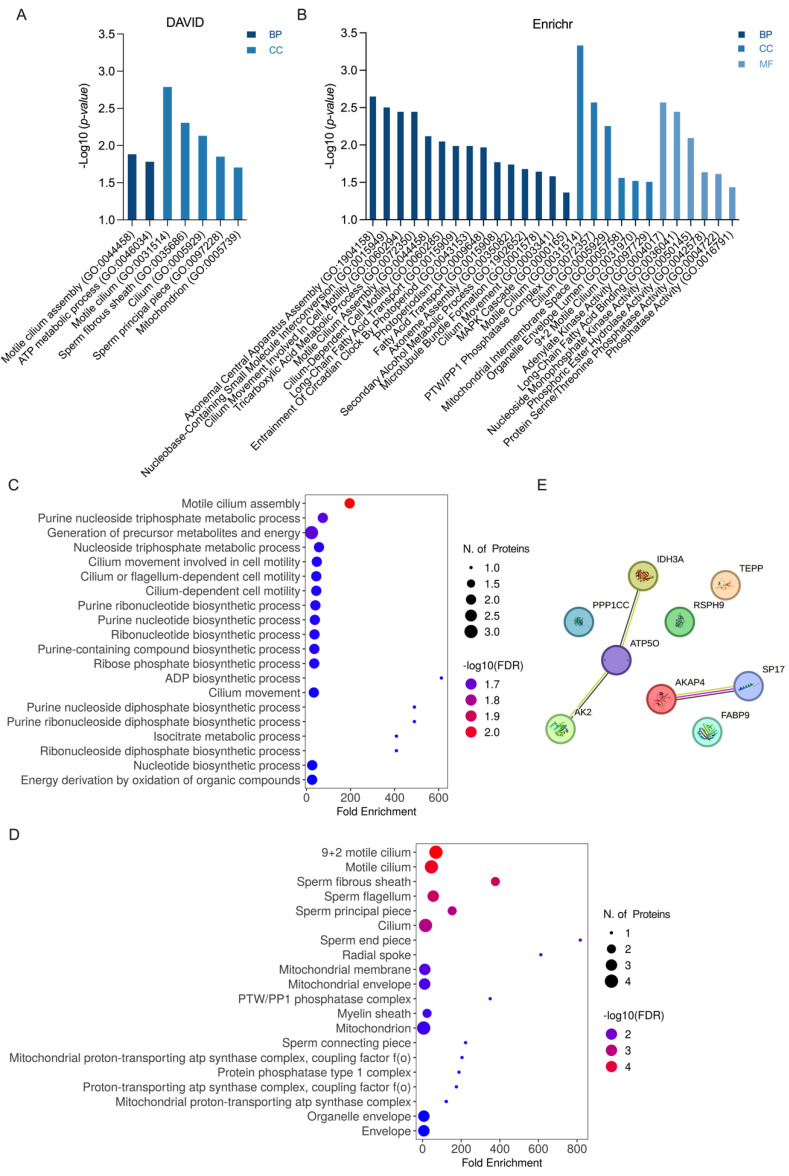
Pathway enrichment and protein–protein interaction (PPI) network between DEPs. Related pathways and cellular processes of DEPs were visualized. (**A**) All enriched categories with *p*-values of < 0.05 were subjected to DAVID GO pathway analysis. Indigo bar = BP (biological process); sky-blue bar = CC (cellular component). (**B**) Enriched all categories with *p* < 0.05 were subjected to Enrichr GO analysis. Indigo bar = BP; sky-blue bar = CC; light-gray bar = MF (molecular function). (**C**) The top 20 enriched BP categories with *p*-values of < 0.05 identified using ShinyGO 0.77 analysis and visualized using a dot plot. (**D**) The top 20 enriched CC categories with *p*-values of < 0.05 identified using ShinyGO 0.77 analysis and visualized using a dot plot. (**E**) PPI network constructed using STRING with nine DEPs in Mus musculus (*p* = 0.017). The confidence cutoff for interaction was set to medium (0.400). Purple edges = experimentally determined associations; green edges = interactions predicted through text mining; black edges = interactions based on co-expression.

The GO annotation and enrichment results obtained from the DAVID database are shown in Figure 5A. The BPs “motile cilium assembly” and “ATP metabolic process” were seen to be significantly enriched, along with five terms related to “motile cilium”. A total of 15 BP terms were categorized, including the most significantly related term, “axonemal central apparatus assembly”, from the Enrichr database (Figure 5B). Furthermore, six enriched terms in the CC category were related to “cilium”, “organelle envelope”, and “protein phosphatase type 1 complex” (Figure 5B). In addition, six enriched terms associated with ‘nucleoside monophosphate kinase activity’, ‘fatty acid binding’, and ‘phosphoric ester hydrolase activity’ were noted in the MF analysis (Figure 5B). ShinyGO analysis presented the top 20 enriched GO terms in BP (Figure 5C) and CC (Figure 5D), and these were consistent with the findings of DAVID and Enrichr analyses. To gain further insights into the cellular processes, PPI analysis was conducted on the DEPs using the STRING database. The PPI network comprised nine nodes and three edges, with a PPI enrichment *p*-value of 0.017. Within the PPI network, three DEPs (IDH3A, ATP5O, and AK2) were found to have direct interactions, as well as two DEPs (AKAP4 and SP17) (Figure 5E).

## 4. Discussion

The extensive usage and increased residual accumulation of the broad-spectrum pesticide bifenthrin pose significant environmental and public health concerns globally. Although there is considerable evidence confirming the overall risks of bifenthrin exposure, there is notably less evidence regarding its effects on male reproductive health, particularly on germ cells. Therefore, the current study examined the toxic effects of bifenthrin on sperm by assessing any dysfunctions in intermolecular mechanisms induced by the pesticide.

The findings of this study showed that bifenthrin generally decreased sperm motility and kinematics. As was stated above, bifenthrin suppresses motility and kinematic parameters commencing at 1 µM treatment (Table 1). These results are consistent with those of Xiang et al. [29], who also reported that bifenthrin decreased total sperm motility but did not affect concentration. These reductions also potentially affect sperm ATP levels, with a previous study reporting an association between sperm motility and intracellular ATP levels [30]. Consistent with the literature, the current study revealed decreased intracellular ATP levels and a positive correlation among ATP level, sperm motility, and kinematic parameters (Table 3). However, the highest correlation coefficient was observed between intracellular ATP and TEPP protein (also known as sperm microtubule inner protein 8) expression (r = 0.868), with a significance lower than 0.01 (Table 3). The current study revealed that 0.1 µM bifenthrin treatment decreased TEPP expression (Figure 3J). 

Previous studies have shown that *Tepp* expression was confined to the testis, prostate, and placenta [31] and can be used as a biomarker for prostate, breast, and ovarian cancers [32]. Recently, Garin-Muga et al. [33] reported that TEPP was detected in human spermatozoa and observed downregulation of the *Tepp* gene expression in equine placentitis disease [34]. Although the function of the TEPP protein appears limited, our results of the correlation analysis were in agreement with those of Satir [35], who found that the microtubules of the sperm flagella use ATP to generate sperm movement. This led to the hypothesis that TEPP proteins, which were localized in doublet microtubules of sperm and not parts of the microtubules only, play a role in sperm flagellar movements using ATP.

On the other hand, no evidence regarding bifenthrin cytotoxicity or direct effects on sperm cell viability was reported in the current study. Previously, Wang et al. [25] reported that bifenthrin at concentrations of 1 and 0.01 µM exerted cytotoxic effects on Hela cells and Chinese hamster ovary cells, respectively, after 72 h of incubation. Despite the higher concentration of bifenthrin exposure compared to previous studies, no evidence regarding cytotoxicity on sperm cells was noted in the current study (Figure 2B), which may be attributed to differences in exposure duration. This speculation is supported by the finding of Wang et al. [25], who reported that bifenthrin did not affect cell viability after 24 h of treatment. However, further studies are warranted to assess sperm cell viability in response to chronic and acute exposure to bifenthrin.

Interestingly, acrosome reaction was abnormally increased by almost 2-fold in bifenthrin-affected sperm compared with the controls (Figure 1B), and this was accompanied by a decrease in the percentage of capacitated sperm. These findings suggest that bifenthrin induced a spontaneous acrosome reaction before interacting with the zona pellucida, thereby decreasing fertilization ability [36].

**Figure 6:**
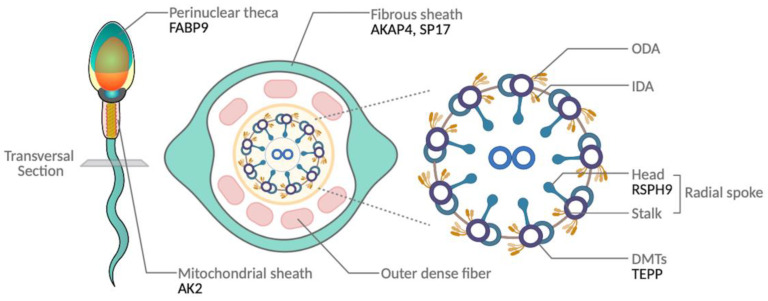
An illustration of the localization of DEPs in sperm. Previously identified localization of DEPs in sperm.

Sperm structure is a fundamental indicator of reproductive ability in both spontaneous fertilization and artificial insemination. Therefore, male fertility is largely dependent on the retention of the structural form of sperm. In the current study, over half of the DEPs identified (i.e., RSPH9, TEPP, SP17, AKAP4, and PPP1CC2) were associated with sperm flagella structure and movement (Figure 5A–D), with RSPH9 and TEPP exhibiting a significant decrease upon exposure to 0.1 µM of bifenthrin (Figure 3J and Figure 4D). RSPH9 is a component of the radial spoke head of the 9 + 2 axoneme assembly and is essential for the maintenance of the central pair of microtubules [37] (Figure 6). Mutations of *Rsph9* have been previously shown to cause primary ciliary dyskinesia with central microtubule pair abnormalities [38]. An SP17 protein—alternative name ‘Sperm autoantigenic protein 17′—has been known to be localized in the testis and fibrous sheath of sperm principal piece in mammals [39] (Figure 6) and participate in the secondary binding of acrosome-reacted sperm and zona pellucida [40]. It plays a role in sperm capacitation [39] and acrosome reaction [41]. In our study, the Western blot analysis observed significantly decreased SP17 expression, and this was in coherence with the 2-DE results (Figure 3F and Figure 4E). Previously, SP17 has been shown to be abundantly expressed in high-fertile buffalo bulls (conception rates: 51% to 56.7%) compared with that in low-fertile bulls (conception rates: 28.8% to 33.8%; log 2-fold change = 2.47) [42]. Consistent with previous research, the findings of the current study showed that low SP17 expression is correlated with decreased fertility parameters, including sperm motility and intracellular ATP levels (Table 3). The 2-DE results showed similar decreasing patterns in AKAP4 and SP17 expression; however, the Western blot analysis showed that SP17 expression decreased to a greater extent even at lower concentrations of bifenthrin when compared to AKAP4. Previously, SP17 has been shown to regulate the AKAP complex in somatic and germ cells [39]. AKAPs bind to PKA, which plays a regulatory role in capacitation and induces signal transduction between the PKA-related intermolecular processes. The findings of the current study showed that both SP17 and AKAP4 exhibited a positive correlation with the percentage of capacitated sperm (r = 0.571 and 0.692, respectively; Table 3). AKAP4, which is localized in the sperm fibrous sheath, has been shown to be associated with sperm flagellar movement [43] (Figure 6). Previously, Miki et al. [44] generated *Akap4* gene knockout mice to examine various sperm defects and found that mice lacking AKAP4 exhibited reduced diameter and length of the principal piece, separated flagella, and decreased sperm motility when compared to the wild-type. Moreover, AKAP4 was highly abundant in the highly fertile buffalo bulls (conception rates: 51% to 56.7%) compared to those with lower fertility (conception rates: 28.8% to 33.8%; log 2-fold change = 3.78) [42]. Recently, Silva et al. [43] reported that AKAP4 in human sperm showed an interaction with PPP1CC2, which was also identified as a DEP in the current study. PPP1CC2 is an isoform 2 of serine/threonine-protein phosphatase PP1-gamma catalytic subunit, a component of the PTW/PP1 phosphatase complex. PPP1CC2 is an alternatively spliced transcript from the *Ppp1cc* gene, and isoform 2 of PPP1CC is a testicular germ cell- and sperm-specific isoform abundantly expressed in the testis [43,45]. In the current study, PPP1CC2 expression first decreased significantly upon exposure to 1 µM bifenthrin and then plateaued (Figure 3E,H). Although the definitive function of PPP1CC2 remains unknown, several studies have reported an association between PPP1CC2 and sperm. Dudiki et al. [46] reported that *Ppp1cc* KO mice showed aberrant sperm morphogenesis, and PPP1CC2 may produce a greater proportion in general sperm function than isoform 1. Moreover, PPP1CC2 is essential for flagella structure and plays a key role in the regulation of sperm motility [45]. Therefore, lacking PPP1CC2 can result in infertility in male mice [46,47]. The current study also revealed a correlation between PPP1CC2 expression and sperm capacitation status, although further research is warranted to elucidate the effects of PPP1CC2 on sperm (Table 3). The PPI network findings revealed interactions between SP17 and AKAP4 (Figure 5E), suggesting an intricate interplay among the three proteins: SP17, AKAP4, and PPP1CC2. Moreover, these three proteins were individually or collectively associated with sperm motility and capacitation (Table 3).

Furthermore, the current study revealed that bifenthrin altered metabolism-related sperm proteins, such as AK2, ATP5O, FABP9, and IDH3A (Figure 5A–D). The expression of AK2 protein showed the most drastic downregulation among nine proteins, and the expression was significantly decreased from a 1 µM treatment of bifenthrin (Figure 3I,L). In sperm cells, AK2 is localized in the mitochondrial sheath and plays a role in energy metabolism [48,49] (Figure 6). Consistent with previous evidence, the findings of the current study showed that intracellular ATP levels were positively correlated with AK2 expression. Moreover, the expression levels of AK2 exhibited the highest correlation coefficient with VSL (r = 0.826, *p* < 0.01). Furthermore, AK2 expression was negatively and positively correlated with acrosome reaction and capacitation status, respectively (Table 3). Future studies are warranted to examine the role of AK2 within the sperm. The 2-DE results showed that ATP5O was decreased only upon exposure to the highest concentration of bifenthrin, whereas Western blot analysis demonstrated that 1 µM bifenthrin suppressed ATP5O expression (Figure 3B and Figure 4B). ATP5O plays a role in oxidative phosphorylation [50], and Rahman et al. [51] reported that ATP5O can regulate calcium ion homeostasis, which is a key event in capacitation. In agreement with these findings, the current study showed that ATP5O expression was positively correlated with the capacitated sperm count (Table 3). Moreover, three of four metabolism-related DEPs (i.e., AK2, ATP5O, and IDH3A) exhibited interactions with ATP5O centrically (Figure 5E). Bifenthrin treatment resulted in statistically significant differences in IDH3A protein expression at concentrations of 1, 10, and 100 µM (Figure 3K). Furthermore, IDH3A exhibited the highest positive correlation with total sperm motility out of the nine DEPs (Table 3). Previous evidence suggests that IDH3A is involved in the TCA cycle and can interrupt sperm motility via energy metabolism [52]. The findings of the current study are also in agreement with [53], who detected a lower expression of IDH3A in asthenozoospermic patients.

FABP9, another metabolism-related protein, is a male germ cell-specific protein that is localized in the perinuclear theca of sperm [54] (Figure 6). As shown in Figure 3A and Figure 4C, the expression of FABP9 decreased significantly upon exposure to higher concentration treatments of bifenthrin (i.e., 10 and 100 µM). Previously, Fabp9−/− mice have been shown to exhibit an 8% increase in sperm head abnormalities compared to the wild-type, although no such effects were observed on sperm count, membrane structure, and fertility [54]. Although the specific role of FABP9 in sperm remains unclear, the findings of the current study show that it exhibits a strong correlation with sperm capacitation (r = 0.853, *p* < 0.01; Table 3).

## 5. Conclusions

In this study, the structural and functional evaluation of bifenthrin-affected spermatozoa was demonstrated. Our preliminary findings demonstrated that although bifenthrin did not affect cell viability, it caused a general decrease in the fertilization ability of sperm. It also altered specific sperm proteins that play a key role in sperm flagella and metabolism. Moreover, the altered protein expression was positively or negatively correlated with various sperm functions, such as motility, capacitation, and intracellular ATP levels. These findings suggest that bifenthrin exposure in mice can result in functional and molecular alternations that are toxic to the fertility potential of sperm. Through this study, we can speculate how sperm function and male fertility are linked to the exposure of bifenthrin. However, the current in vitro study only focused on direct mechanisms of action on sperm. Further in vivo studies with different periods of exposure are suggested.

## Figures and Tables

**Table 1:** Sperm motility and kinematics after bifenthrin treatment.

Parameter	Concentration (µM)				
	0	0.1	1	10	100
Motility					
MOT	71.86 ± 0.70 ^a^	66.06 ± 3.24 ^a,b^	59.84 ± 2.78 ^b,c^	57.87 ± 3.48 ^b,c^	52.92 ± 2.50 ^c^
PRG	71.21 ± 0.81 ^a^	65.45 ± 3.25 ^a,b^	59.56 ± 2.87 ^b,c^	57.73 ± 3.49 ^b,c^	52.70 ± 2.48 ^c^
HYP	18.23 ± 2.83 ^a^	13.40 ± 2.33 ^a,b^	13.78 ± 1.66 ^a,b^	10.92 ± 1.72 ^a,b^	6.90 ± 1.41 ^b^
Kinematics					
VCL	74.93 ± 3.06 ^a^	65.04 ± 5.10 ^a,b^	61.99 ± 5.81 ^a,b^	56.57 ± 7.43 ^a,b^	49.76 ± 4.61 ^b^
VSL	29.44 ± 2.43 ^a^	24.09 ± 2.29 ^a,b^	23.37 ± 1.66 ^a,b^	20.55 ± 2.01 ^a,b^	15.36 ± 1.94 ^b^
VAP	42.05 ± 2.40 ^a^	35.43 ± 2.82 ^a,b^	33.55 ± 2.56 ^a,b^	30.18 ± 3.34 ^a,b^	24.91 ± 2.63 ^b^
LIN	30.09 ± 1.02 ^a^	25.88 ± 1.80 ^a,b^	23.94 ± 0.72 ^b^	22.77 ± 0.64 ^b,c^	18.17 ± 1.02 ^c^
STR	69.52 ± 2.15 ^a^	67.27 ± 1.91 ^a,b^	69.34 ± 1.17 ^a^	67.84 ± 0.85 ^a,b^	60.62 ± 3.04 ^b^
BCF	5.51 ± 0.23 ^a^	4.77 ± 0.35 ^a,b^	4.68 ± 0.47 ^a,b^	4.15 ± 0.58 ^a,b^	3.61 ± 0.38 ^b^
ALH	2.99 ± 0.07 ^a^	2.67 ± 0.19 ^a,b^	2.50 ± 0.22 ^a,b^	2.29 ± 0.28 ^a,b^	2.11 ± 0.18 ^b^

The sperm motility and kinematic values are presented as the mean ± SEM (*n* = 4). MOT = total sperm motility (%); PRG = progressive sperm motility (%); HYP = hyperactivated sperm motility; VCL = curvilinear velocity (μm/s); VSL = straight-line velocity (μm/s); VAP = average path velocity (μm/s); LIN = linearity [%, (VSL/VCL) × 100]; STR = straightness [%, (VSL/VAP) × 100]; BCF = beat cross frequency (Hz); ALH = mean amplitude of head lateral displacement (μm). Superscript letters (a, b, and c) indicate significant differences between the control and each treatment group (*p* < 0.05).

**Table 2:** Differentially expressed (>3-fold) proteins identified via LC–MS/MS.

Symbol	Accession	Description	Score *	MW
FABP9	O08716	Fatty acid-binding protein 9	2857	15,236
ATP5O	Q9DB20	ATP synthase subunit O, mitochondrial	1812	23,406
AKAP4	Q60662	A-kinase anchor protein 4	785	95,559
PPP1CC2	P63087-2	Isoform 2 of Serine/threonine-protein phosphatase PP1-gamma catalytic subunit	770	39,221
SP17	Q62252	Sperm surface protein Sp17	277	17,342
RSPH9	Q9D9V4	Radial spoke head protein 9 homolog	123	31,368
AK2	Q9WTP6	Adenylate kinase 2, mitochondrial isoform b	92	25,817
TEPP	Q6IMH0	Testis, prostate, and placenta-expressed protein isoform 2	85	12,510
IDH3A	Q9D6R2	Isocitrate dehydrogenase (NAD) subunit alpha, mitochondrial	62	40,069

* Protein score is −10 log (p), where p is the probability that the observed match is a random event. Individual scores greater than 55 are considered significant (*p* < 0.05).

**Table 3:** Correlation between sperm parameters and expression of DEPs.

	MOT	PRG	HYP	VCL	VSL	VAP	BCF	ALH	AR	B	F	ATP	Viability	FABP9	ATP5O	AKAP4	PPP1CC2	SP17	RSPH9	AK2	TEPP	IDH3A
MOT	1	0.999 **	0.776 **	0.929 **	0.895 **	0.939 **	0.911 **	0.920 **	−0.644 **	0.878 **	−0.306	0.607 **	−0.248	0.727 **	0.566 *	0.519 *	0.691 **	0.394	0.723 **	0.760 **	0.769 **	0.806 **
PRG		1	0.782 **	0.936 **	0.899 **	0.944 **	0.920 **	0.927 **	−0.642 **	0.876 **	−0.306	0.595 **	−0.240	0.719 **	0.557 *	0.514	0.679 **	0.385	0.720 **	0.750 **	0.760 **	0.793 **
HYP			1	0.850 **	0.936 **	0.908 **	0.834 **	0.805 **	−0.723 **	0.867 **	−0.194	0.549 *	0.217	0.669 **	0.488	0.377	0.616 *	0.357	0.755 **	0.691 **	0.783 **	0.685 **
VCL				1	0.909 **	0.965 **	0.989 **	0.984 **	−0.607 *	0.820 **	−0.279	0.464 *	−0.163	0.616 *	0.439	0.380	0.561 *	0.226	0.624 *	0.650 **	0.679 **	0.594 *
VSL					1	0.981 **	0.912 **	0.844 **	−0.731 **	0.927 **	−0.260	0.582 **	0.047	0.768 **	0.596 *	0.544 *	0.729 **	0.454	0.719 **	0.826 **	0.852 **	0.794 **
VAP						1	0.964 **	0.922 **	−0.703 **	0.891 **	−0.248	0.568 **	−0.046	0.730 **	0.568 *	0.480	0.687 **	0.358	0.708 **	0.800 **	0.805 **	0.751 **
BCF							1	0.963 **	−0.548 *	0.791 **	−0.315	0.443	−0.206	0.595 *	0.372	0.383	0.528 *	0.184	0.527 *	0.672 **	0.670 **	0.548 *
ALH								1	−0.575 *	0.768 **	−0.253	0.479 *	−0.225	0.566 *	0.362	0.298	0.506	0.157	0.624 *	0.583 *	0.623 *	0.548 *
AR									1	−0.682 **	−0.385	−0.872 **	−0.425	−0.704 **	−0.490	−0.307	−0.572 *	−0.508	−0.795 **	−0.662 **	−0.841 **	−0.732 **
B										1	−0.412	0.651 **	−0.032	0.853 **	0.633 *	0.692 **	0.725 **	0.571 *	0.676 **	0.707 **	0.829 **	0.834 **
F											1	0.265	0.522	−0.199	−0.188	−0.491	−0.203	−0.088	0.138	−0.068	0.002	−0.141
ATP												1	0.350	0.819 **	0.491	0.404	0.693 **	0.570 *	0.786 **	0.735 **	0.868 **	0.853 **
Viability													1	0.050	0.102	0.048	0.054	0.487	0.343	0.136	0.035	−0.028
FABP9														1	0.672 **	0.680 **	0.736 **	0.667 **	0.672 **	0.619 *	0.868 **	0.868 **
ATP5O															1	0.619 *	0.535 *	0.585 *	0.514	0.418	0.488	0.677 **
AKAP4																1	0.462	0.853 **	0.231	0.382	0.442	0.567 *
PPP1CC2																	1	0.362	0.748 **	0.824 **	0.681 **	0.862 **
SP17																		1	0.385	0.279	0.503	0.532 *
RSPH9																			1	0.669 **	0.692 **	0.810 **
AK2																				1	0.737 **	0.791 **
TEPP																					1	0.825 **
IDH3A																						1

MOT = total sperm motility (%); PRG = progressive sperm motility (%); HYP = hyperactivated sperm motility; VCL = curvilinear velocity (μm/s); VSL = straight-line velocity (μm/s); VAP = average path velocity (μm/s); BCF = beat cross frequency (Hz); ALH = mean amplitude of head lateral displacement (μm); AR = acrosome reacted sperm; B = capacitated sperm; F = non-capacitated sperm; ATP = intracellular ATP levels; Viability = cell viability. * Correlation is significant at the 0.05 level (2-tailed). ** Correlation is significant at the 0.01 level (2-tailed).

## Data Availability

All the data generated or analyzed during this study are included in this published article.
